# Democratic Deterrence of Middle Powers in Great Power Rivalry: The Case for Indonesia

**DOI:** 10.12688/f1000research.169856.2

**Published:** 2025-12-31

**Authors:** Aristo Purboadji

**Affiliations:** 1 Theology, Sekolah Tinggi Teologi Tiberias Jakarta, Central Jakarta, Jakarta, 10150, Indonesia

**Keywords:** Great Power Rivalry; Middle Powers; Democratic Advantage Argument; Democratic Deterrence; Emancipative Values

## Abstract

Shortly after the Russian invasion of Ukraine, President Biden rightly characterized the current era of great power competition as one that occurs between democracies vs autocracies; thus, democracies need a new kind of deterrence concept against emboldened autocrats, as exemplified in Mikael Wigell’s ‘democratic deterrence’ that calls for greater apprehension of –and confidence in—democratic advantage argument in the public consciousness. Democratic middle powers in general could play an important part in the aforementioned democratic deterrence in their own respective capacities, and the third largest democracy, in particular, has the potential to play a unique role in promoting a democratic advantage narrative, especially in the case of the compatibility of democracy with Islam through the principle of *Maqashid Sharia*. This study makes three recommendations for Indonesia to fulfill its potential role. First, Indonesia needs to accelerate its democratic consolidation process by raising the public’s emancipative values, especially through cognitive mobilization but allied with religions. Second, democratic advantage apprehension must be integrated within traditional foreign policy principles, as the two are not at all contradictory. Finally, the West in general, and United States in particular, need to be more appreciative and supportive of Indonesia’s effort to play a middle-power stabilizing role in the Indo-Pacific, whether in the forms of economic or defense cooperation.

## Introduction

The Russian invasion of Ukraine adds to the growing list of Putin’s aggression besides meddling in the US election, annexation of Crimea and Georgia, bloody intervention in Syria, and countless disinformation campaigns. Just six days after the invasion, President Biden expanded Trump administration doctrine of ‘great power competition’ to ‘battle between democracy and autocracy’ in the State of the Union address, raising great power competition to a new level. Days before the actual invasion to Ukraine, China and Russia declared a ‘no limit’ partnership on the opening day of the Winter Olympics, raising speculation of China’s foreknowledge of – and blessing for—the coming invasion.

Indeed great power politics have been seen as the central stage in the field of international history ( Iriye, 2013, p. 6). Great power politics and the polarity it causes is deemed as “an essential starting point for thinking about international relations.” ( Buzan, 2004, p. 31) Waltz (1979, pp. 72-73) states that “general theory of international politics is necessarily based on the great power.” Combining the discussion about great powers with the battle between democracy and autocracy makes things more interesting.

The autocracy vs. democracy dichotomy of today’s geopolitics stresses that the new era of great power rivalry is not just in the domains of military, economy, diplomatic or technology, but it is structurally ideological. The United States champions democratic governance, human rights, free speech, and free-market capitalism. Russia’s Putinism is basically a kleptocracy that aims to resurrect the Russian empire and multipolar world order, while China’s Chinese Communist Party (CCP) blends one-party rule with state-led capitalism.

The growing Russian aggression and China’s assertiveness has one particular backdrop narrative that many suspect as the reason for the emboldened autocrats: the decline of the democratic West. Scholars dated China’s peaceful rise diplomatically from 1991 to 2008 ( Kang, 2007) and has somewhat changed ever since the Global Financial Crisis of 2008 ( Khoo, 2022, p. 145) the same year, Fareed Zakaria published his book “The Post-American World” ( Zakaria, 2008). Indeed, the 2008 financial crisis is a watershed moment in modern history, reshaping perceptions of American economic prowess and global leadership. It exposed vulnerabilities in the American economic system and eroded trust in the free-market capitalism model; many perceived it as contributing to a shifting global balance of power. It is the pivotal moment that “… convinced many Chinese that American’s decline has truly and finally come” ( Shambaugh, 2013, p. 19) and created a sense of triumphalism among the Realists in China that state-led capitalism is vindicated and *laissez faire* has been vanquished (pp. 27-28). Interestingly, it was during the period of the aforementioned financial crisis, in 2009 to be exact, that “… the map with nine-dash line was attached in a submission to the U.N. during a dispute with Vietnam” ( Beech, 2016). Since then Xi Jinping’s regime abandoned the guiding philosophy of Deng Xiaoping’s foreign policy “Hide your strength and bide your time” since the CCP has felt that its moment has arrived with the West in decline.

In essence, the decline of the West or American declinism is related to the perceived decline of democracies. According to Ignatius Wibowo, a prominent Indonesian scholar, the global financial and economic crisis, “… has increased the attractiveness of the Chinese model of authoritarian capitalism for some emerging markets” and this has indeed “… added to democracy’s setbacks” ( Kurlantzick, 2013). The 2008 global financial crisis is not the only symptom that has been pointed out as proof of the structural flaws in the US economy. Income inequality and the erosion of the middle class have been added to the list. The US governability and political system have also been questioned with growing political polarization, epitomized by the January-6 storming of Capitol Hill. The withdrawal of American forces from Afghanistan concluded with the swift takeover of the country by the Taliban seems to add weight to the perceived decline militarily.

2008-2010 global financial crisis also was the period where the upsurge of writings about ‘China model’ has started ( Shambaugh, 2013, p. 28) leading to its increasing nationalism and assertiveness in the region (p. 31) and “… triggering much euphoria and vindication in China concerning its state/market development model” (p. 171), a model depicted by Cooper Ramo as “… unique blend of authoritarian politics and a mixed state/market economic model offered the developing world an appealing alternative to the “Washington Consensus” of democracy and free market capitalism” (p. 171). One confident Tsinghua University professor even declared the possibility that the Beijing Consensus will replace the Washington Consensus ( Kurlantzick, 2013).

Adding to the aforesaid advance of ‘China model’ is Putin’s ambition to resurrect greater Russia ( Socor, 2018) that has been demonstrated in 2022 invasion to Ukraine. A senior Chinese expert on Russia remarked that Russia is the most important country for China diplomatically, and a growing number of officials in foreign policy believe that Moscow is China’s real strategic partner and not Washington ( Shambaugh, 2013, p. 29). In this daunting two-pronged competition some scholars predict that this American decline will return the world to a multipolar balance of power systems, where each great powers leads its own respective region ( Kupchan, 2012). Others predict bleaker outcomes when China takes over global leadership from United States ( Jacques, 2012). Worst of all, some have predicted the inevitability of World War III ( Allison, 2017).

We can agree with Brands (2018, p. 61) when he points out “… that modern great-power competition revolves around clashes of ideologies and systems of government no less than around clashes of national interests”. Friedman (2009a) for one lauds autocratic system for being able to take decisive action and do not have to get drowned in endless debate, even including a chapter titled “China for a Day.” Further, Friedman ( The New York Times, 2009b) argues that one-party autocracy, when led by an enlightened group of people, can “… impose the politically difficult but critically important policies needed to move a society forward in the 21 ^st^ century”. Interviewed in *The Colbert Report* he explained that “China for a Day” is a fantasy if America has a government that can actually make decisions, make long-term plans, and pursue it ( Center for Individual Freedom, 2008). China’s seemingly superior system of governance can be seen in several works of Western intellectuals, such as Harvard University professor and sociologist Daniel Bell, whose discussion of the China model’s strong emphasis on political meritocracy and state-guided development, outlining some strengths that the China model possesses, such as political stability and efficient centralized decision-making ( Bell, 2015).

More worryingly, Russia and China are keen to export their autocratic models, ever ready to usher in an increasingly authoritarian world. Kremlin’s strategies include supporting nationalist and anti-Western movements in various countries, propagating state-controlled media, supporting like-minded leaders such as Belarusian President, Alexander Lukashenko, and installing puppet regimes such as Ukrainian previous President, Viktor Yanukvych.

Edel and Shullman (2021) explain three broad categories of China’s international efforts to subvert democracy: 1) Attempts to shape the narrative about China in developed countries, 2) Share China’s narrative to developing countries, and 3) Weakening international institutions and creating new ones that are non-democratic. Measures taken in the second category have been more successful than the first, proving that the developing world and the Middle Powers are more vulnerable to China’s autocratic influence, although the most recent China’s interference in Canada’s elections wreaked many havocs ( Austen, 2023). The list of China’s interference in Australia is massive, with scandals such as the Labor Senator Sam Dastyari, who was forced to step down over undeclared payments from several People’s Republic of China (PRC) businessmen, former Defense Minister accepting paid travel to China, donations to political parties from pro-CCP tycoons, and numerous others ( Chubb, 2023, pp. 21-22). CCP-linked organizations, for example, shared their surveillance technology and its Great Firewall with countries such as Uganda and Zambia. In the third category, China, for instance, wielded its influence in the International Telecommunication Union to promote policies conducive to authoritarian practices ( Edel & Shullman, 2021).

The vulnerability of Association of Southeast Asian Nations (ASEAN) countries to the appeal of China model is put forth by the aforementioned Ignatius Wibowo, concluding that “… people in Southeast Asian share a willingness to abandon some of their democratic values for higher growth, and the kind of increasingly state-directed economic system that many of these countries had, in their authoritarian days, and that China still has today”. Southeast Asian nations shifted their democratic development strategy to one “… based on semi-free markets and an illiberal political system” with measures such as enhancing the state control of strategic industries, centralizing political decision-making, weaponizing judicial system as the tool of the state (using judicial rulings to weaken opposition parties), Internet monitoring and blocking, and strengthening one-party domination. Further, according to Wibowo, the political trajectory of ten states in the ASEAN has regressed back a decade because “… these nations had watched China’s successes and contrasted it with the West’s failures” ( Kurlantzick, 2013).

Germany’s call for a new ‘Marshall Plan’ for democracy cannot be overstated ( Kleinfeld et al., 2021, p. 7). This paper addresses two questions: first, how can middle powers be a part of democratic deterrence in the era of great power rivalry, and second, how can Indonesia play a role in such deterrence? The global stage has indeed become a theatre where the Chinese model and the liberal democracy model are under scrutiny. The ongoing observation of these competing contrasting models will shape the trajectory of global governance in the years to come. In this great power rivalry of autocracy and democracy, the rest of the world is watching, including the Middle Powers.

## Counter-argument against democratic decline

The battle between autocracy and democracy is ideological in nature, hence, it is a battle among competing values that must be won, not just physically but also intellectually. The most important element of an effective intellectual deterrence for democracy is the democratic advantage argument: How can one convince another for the superiority of the democratic system compared to the other? Perhaps the most comprehensive and convincing intellectual work for the democratic advantage argument is Matthew Kroenig’s democracy advantage argument formulated in his book “The Return of Great Power Rivalry” ( Kroenig, 2020). Kroenig himself is on the list of Americans sanctioned by Russia following the recent invasion of Ukraine, along with figures like Mark Zuckerberg and U.S. Vice President Kamala Harris ( Fortune Magazine, 2022). Democratic advantage argument doesn’t only advancing moral case for democracy but also the “hard-power” argument for democracy: Democracy is better in creating wealth, making diplomatic alliance, even dominate in military and war.

The recent rise in global populism and polarization globally should be a wake up call for democracy survival. Populist candidates with autocratic tendencies are gaining ground in polls and elections worldwide. Russian support of far-right parties in Europe (such as Marine Le Pen’s National Rally in France) is a well-known fact and proves that autocrats stoke polarization by amplifying societal divisions, whether along ethnic, religious, or economic lines. Naim (2022) explains that the rise of populism and the backsliding of democracy have made the majority of world population now live in autocratic countries. The appeal of autocracies, be it the ‘China model’ or Putin’s kleptocracy, needs potent intellectual countering. Kroenig’s “The Return of Great Power Rivalry” could be the countering needed. Previous studies on the superiority of democracy have been conducted in a wide range of discrete areas, such as economic growth ^1^, alliances ^2^, or military power ^3^. What is novel with Kroenig’s work is that he aggregates these narrower findings into argument of democracy’s superior great-power-long-run competitive advantage over autocracy. He deems his work as empirical, as 2500 years of history supported his claim that a more open society tends to excel in great power competition, drawing from the Ancient World to the Cold War, where seven cases of democracy vs. autocracy are presented. Combined with this empirical proof, is the reservoir of the political philosophy canon from Herodotus to Montesquieu and cutting-edge social research that all point towards democracy superiority over autocracy. Kroenig’s democratic advantage argument is countering against what Kroenig calls as ‘Contemporary Autocratic Advantage Theory’ with arguments such as: Autocrats are ruthless decision makers that are not bound by public opinion and ethical reasons; Autocrats can make long-term strategic plan and pursue it; Autocrats can take big, bold decisions whereas democracies dither in endless debate; and lastly, Autocrats has clean, stable and efficient politics. These arguments behind the logic of China model’s and Putinism’s appeal are effectively neutered by Kroenig’s democratic advantage thesis.

Lastly, Kroenig predicts the still-autocratic Russia and China in the future, and they are not at all sunshine and rainbows. Russia’s and China’s economic, diplomacy, and military power all suffered from acute structural flaws for the very reason that they were autocratic. There were talks about China’s economy surpassing that of the United States by 2020, and economists now delay it to 2040. But Kroenig (2020, p. 183) predicts that ‘never’ is also the likely outcome for reasons such as: Chinese leaders increasing autocratic economic policy that choose political control over economic efficiency; declining population combined with strict immigration policy; technology Intellectual Property (IP) theft practice that led to decoupling policy of U.S. In conclusion, in the absence of market reforms that require the more inclusive economic policies, China’s export-led growth model will run out of steam. There is also doubt whether China’s technology industry, particularly its Artificial Intelligence (AI)’s prowess, can live up to the hype. AI requires microchips, a crucial technology that China lags behind, and with a trade ban and U.S. decoupling policy, its industry will suffer setbacks. China excels in certain applications of AI such as facial recognition and creating algorithms to spy on its citizens, but such technology is not desired by major world economies, so simply the demand for the product is weak. Further, its policy of civil-military fusion creates suspicions and distrust for China’s product, as evident in the ban on Huawei’s affordable 5G wireless technology by United States, followed by other countries. The autocratic tendency to sacrifice progress over control is exemplified in Russia’s ambition to build its own version of the Great Firewall by installing the Internet watchdog ‘Roskomnadzor’ to block sites and interrupt networks and in its tech indigenization ambition. Putin is restarting a failed Soviet-era ambition to create the Russian Silicon Valley in Zelenograd near Moscow, with doubtful results. The overall performance of the technology industry is meager in sectors such as semiconductors (Baikal and Mikron), software (ABBY and Kaspersky), cloud (amoCRM, Miro, and New Cloud Technologies), and social media (VK) ( The Economist, 2022).

## Democratic deterrence & middle powers

There have been scholarly discussions about the need for democratic deterrence, whether internally or externally. What I mean by ‘internally’ is the need to deter unscrupulous politicians from using legal machinations to subvert democracy, as shown in the work of Helmke et al. (2021). Some works, such as Clare (2013), discuss the deterrent value of democratic alliance-building ability. Wigell (2021) introduces the concept of ‘democratic deterrence’: “It asks what deterrent value democracy itself has and envisages a host of non-military means to adapt deterrence to the current non-military challenges” (p. 50). Wigell claims novelty in this concept of democratic deterrence in that it dissuades hybrid interference activities by authoritarian states and is different from traditional military deterrence in these manners: 1) The former rests on the whole-of-society approach while the latter is state-based; 2) The former relies on soft power while the latter relies on hard power; 3) The former relies on non-military means while the latter military means; 4) The former tends to induce an asymmetrical response while the latter is symmetrical; and 5) The former realistically accepts the limits of its deterrence, while the latter tends to wholly deterring any aggression.

Like any other deterrence, democratic deterrence strategy is two-pronged or divided into two broad categories: denial (i.e., resilience) and punishment (i.e., compellence). Measures of denial include: 1) activating civil society, 2) increasing transparency, and 3) broadening inclusion. Measures of punishment include: 1) communicate response thresholds, 2) expand sanctions; and 3) promote democracy. From such measures of democratic deterrence we can see that “… democratic values are not only vulnerabilities, but they can be turned into strengths and tools for a credible deterrence response to hybrid interference” (p. 64). Kroenig (2020, p. 32) sums this well:

“Some see the constraints on government power in democracies as a weakness, but, in fact, they are democracy’s greatest strength. These constraints facilitate economic growth by giving individuals and businesses confidence that they will be able to reap the rewards of their labors, investments and innovations. They help democracies attract capital and develop as financial centers, because investors know their money will be safe and will likely generate positive rates of return over the long run. They make for a stronger alliances and international diplomacy, because democratic commitments tend to be more reliable and because others have less to fear from powerful democracies. They lead to superior military performance; since people do not fear their governments, their governments need not fear them, and governments can focus on external enemies. They innovate in military technology and operational concepts. In addition, protections for free speech lead to open debate that informs democratic leaders in foreign policy, including on matters of war and peace”.

It is a mistake to consider democratic principles and norms (such as free speech, inclusion and tolerance) as mistakes. They might be constraints but certainly not mistakes. The strength of democracy inheres in itself. Indeed, democracy is its own deterrence. Doubling down on democratic values and principles such as transparency, inclusion, equality, check and balance is the best deterrence against autocratic attacks: whether external or internal; whether hybrid warfare or hybrid interference; whether intellectual or physical.

This article follows Teo (2021, p. 4)’s definition of middle power as a country that:

“Quantitatively ranks below the great and rising powers, but above a majority of the rest of the states; identifies and is regarded by others as a middle power; and, employs behavioural strategies such as investing in multilateralism and relying on persuasive or soft power”.

Conceptually Teo (2021) describes Middle Powers vertically in terms of their material/quantitative capabilities and horizontal/functional capabilities. Vertically, middle powers possess medium material capabilities, while functionally they are expected to adopt diplomatic practices that rely on multilateralism and dialogue, which are typical of a middle power. While previous works unfairly seem not to place (acknowledge) Indonesia as a middle power, later works increasingly include it ^4^.

Kleinfeld, et al. (2021) defines middle-power democracies as “countries which regardless of their geopolitical weight have made democracy support a sustained component of their foreign policy” and deem them crucial in renovating global democracy support strategy that was perceived as weakening especially during Trump presidency. Measures taken by middle-power democracies to revamp international democracy support include: 1) Enhancing solidarity, 2) Sharpening their focus (i.e., target policy areas aligned with democratic values), and 3) Improving diplomatic cooperation. These measures are necessary for middle-power democracies to counter the growing influence of China and Russia, while avoiding direct confrontation. Five particular strategies are recommended: 1) Pursue democracy-adjacent issues; 2) Leverage regional “swing state” status; 3) Use Track II diplomatic and legislative channels to pursue more controversial policies; 4) Channel economic tools to enhance democratic alliances; and 5) Revive the narrative of democratic economic development to counter one of the most potent areas of authoritarian attraction (pp. 1-2).

Paris (2019) discusses the potential of middle powers to save the liberal international order to prevent its disintegration or at least slow down its erosion from major-powers-autocratic influence, such as China and Russia. Some measures recommended to that end are: 1) defining middle powers’ priorities, 2) assembling issue-specific coalitions with clear goals, and 3) coordinating middle powers’ efforts effectively. Further, Paris (2019) suggests that issue-specific coalitions could consist solely of states, as well as non-governmental organizations (NGOs) such as corporations, private foundations and advocacy networks. We can readily see that this approach is identical to Wigell (2021)’s whole-of-society approach to democratic deterrence.

Combining Wigell (2021)’s concept of ‘democratic deterrence’ with Paris (2019)’s and Kleinfeld (2021)’s middle-power democracies’ role to save global democracy lends us an interesting insight of the potential role that middle-power democracies can play for global democratic deterrence. Kleinfeld (2021)’s pursuit of democracy-adjacent issue strategy and Paris (2019)’s ‘issue-specific coalition’ hint to the specific unique role each respective middle power can play according to their own competitive advantage to help the advance of Kleinfeld (2021)’s fifth autocratic-countering strategy: Revive the narrative of democratic excellence. For example Canada, a country known for its public administration prowess, has developed programs and initiatives to train officials in Central America and the Caribbean to develop governance capacity building, such as budgeting and revenue collections ^5^. Another example is The United Kingdom, known for its expertise in good governance in the mining industry, which helped the establishment of the Extractive Industries Transparency Initiative and became a gold standard in extractive accountability ( Kleinfeld et al., 2021, p. 16).

One specific issue where middle powers can counter autocratic expansion is in the field of ‘digital repression’ which is defined as: “… the burgeoning use of digital technologies to degrade or disrupt democracy—from online disinformation to manipulate elections to the use of artificial intelligence and big-data methods to surveil and repress peaceful protesters” ( Kleinfeld, et al., 2021). One important venue to push back China’s ‘cyber sovereignty’ ^6^ agenda is in the area of regulation, one that European Union has the competitive edge of. The European Commission has proposed policies regulating: internet companies operation in Europe, political ads conduct around disinformation, and surveillance technology export control (such as facial recognition). EU’s initiatives such as the Digital Services Act and the European Democracy Action Plan are promising and can be seen as deterrence against the use of technology by autocratic agents to encroach on democracy. “Paris Call for Trust and Security in Cyberspace”, Microsoft’s call for Digital Geneva Convention and Siemen’s Charter of Trust are some good starts in the domain and can be seen as middle powers’ example of whole-of-society democratic deterrence in the digital area, especially when we consider the impasse of United Nations’ (UN) Groups of Governmental Experts’ (GGE) on cyber and digital matters. To address technological issues, the United Kingdom proposed the creation of the D10 (G7 plus some democracies). Kleinfeld et al., (2021) explains how some middle powers have taken leadership roles in certain sphere of their respective competitive edge, such as the United Kingdom in the anticorruption domain; Canada and Sweden in the domains of injustice, inclusion, and development policies; Australia in the origin and handling of the pandemic; and South Korea in global health security.

Some notable middle-power led democracy-adjacent-issue coalition are: Canada-led “Ottawa Group on WTO Reform,” “Muskoka Initiative on Maternal, Newborn and Child Health,” “Lima Group”; Norway and Canada-led “Human Security Network”; Global Compact for Migration; France and Germany-led Alliance for Multilateralism; France, Germany, Italy, and Netherland-led “The Inclusive Vaccines Alliance”; Sweden-led “Friends in defense of Democracy” and “Drive for Democracy”; “Aarhus Convention” and “Escazu Agreement” to improve democracy in Latin American; “The Halifax International Security Forum” on security and defense issue; Cartel Project to protect journalist; “Inter-Parliamentary Alliance on China” to empower the legislative branch; and Bali Democracy Forum.

Five overlapping points are worth noting when discussing the potential of middle powers to revamp global democracies. First, the importance of including non-Western nation in order to showcase democracy’s success in non-Western world ( Kleinfeld et al., 2021, p. 8) hence Bali Democracy Forum (BDF)’s depiction as the compatibility of “Asian values” with democratic values (p. 20). Second, it is more effective to not directly address democracy, as works of this sort “… will gain buy-in from a wider range of countries than conventional democracy work” (p. 14). Hence, the “democracy-adjacent issues.” It should be understood that when countries advance issues of human rights (e.g., supporting women’s access to justice), they also simultaneously build a democratic muscle. Third, it is important to show the democratic advantage narrative especially in the face of China’s advance of its ‘China’s model’ (p. 20), that serves “… to counter one of the most potent areas of authoritarian attraction” (p. 2). Fourth, the definition of middle-power democracies itself as a country that made democracy support a sustained component of its foreign policy (p. 1); thus, we can argue that this is what Paris (2019) means when he refers to “like-minded countries” (p. 4). Fifth, middle-power democracies should capitalize on their position as regional swing states ( Kleinfeld et al., 2021, p. 19). It is interesting to note that Indonesia is considered as “global swing state” ^7^.

## Democratic deterrence and values

We can argue that the greatest strength of democratic deterrence is its own values. To reiterate, Wigell (2021)’s ‘democratic deterrence’ focuses on democratic norms, principles and values as its greatest strengths. Democratic values like ‘inclusion’ and ‘openness’ could be seen by autocratic agents as weakness, provides them an opening to attack, but it is actually the strategic soft power that exercises a strong international pull (p. 53) and itself “can be a means of compellence” (p. 60). Democratic values “… are not only vulnerabilities, but they can be turned into strengths and tools for credible deterrence response to hybrid interference”. Doubling down on democratic values and principles will only strengthen democracy itself against internal and external attacks:

“Democratic deterrence focuses on strengthening our liberal democratic values and infrastructure: transparency, accountability, inclusiveness, and civil society. To this end, deliberately focusing on *democratic* deterrence will simultaneously improve democratic governance, making Western democracies more robust and resilient” (p. 64).

By focusing on external ‘hybrid interference’ attacks, we can argue that Wigell (2021)’s democratic deterrence is also a deterrence against internal attacks on democracy, such as elected populist politicians’ efforts to undermine domestic democracy. For example Helmke, et al., (2021)’s argument about democratic deterrence on internal democratic backsliding, such as when populist politicans using democracy itself to subvert it (for example with legal machinazation or election distorting) is relying on specific essential norm which is ‘mutual forbearance.’ Thus, norms, principles, and values are essential for democracy to ward off internal and external attacks, since any good constitution without sufficient norms and values can still create “constitutional hardball.”

To appreciate the link between values and the system of governance, it is instructive to reference Welzel (2021)’s “cultural theory of autocracy vs. democracy”. The theory successfully explains how the culture of a given country determines its preference for the governance system, and this explanation has remained constant for the last 120 years, explaining regime change as a corrective response to regime-culture misfits. The explanatory power of the aforementioned explanation rests in the cultures’ differentiation on “authoritarian-vs-emancipative values” where autocracy is described as having authoritarian values such as: coercion, favoritism and clientilism; and democracy is decribed as having emancipative values such as: liberty, impartiality, and contractarianism. The prevalent mentality mode of autocracy in this theory is described as ‘intimidation’ whereas the mentality mode of a democracy is ‘encouragement.’ More about this emancipative values:

“The cultural theory of autocracy-vs-democracy sees the driving psychological force of global regime evolution in the generational rise of emancipative values. These values idealize *universal* human freedoms and combine a *libertarian* emphasis on individual choice with an *egalitarian* emphasis on equality of opportunities. Philosophically speaking, these are essentially the values that inspiried the Enlightenment and the modern reinvention of democracy” (p. 994)

Against the backdrop of the currently trending democratic deconsolidation thesis, Welzel (2021) predicts that the ubiquitous ascencion of emancipative values will lend to more democratization and thus less autocratization, since democracy has a reproductive advantage over autocracy. Further, he boldly hypothesizes that against the current deconsolidation climate, democracy is to “… recover from its current intercession and to bounce back mightily” (p. 994). This is due to one important factor: generational evolution in most regions of the world from authoritarian values to emancipative values. Consequently, we need to find out what determines these potent emancipative values. Welzel gives credit to ‘cognitive mobilization’ as the factor determining the rise of emancipative values: “… the sweeping worldwide expansion of education and other cognition-, awareness- and intelligence-lifting consequences of modernization” (p. 994). This revolution in global cognitive ability, in turn, came about as a result of mankind enabling living conditions. In other words, this cognitive mobility is presented by expanding existential opportunities that happen not in a top-down manner as a result of a fabricated ideology but evolve naturally bottom-up (p. 999). Determinining which comes first between emancipative values and cognitive mobility is a kind of chicken and egg question, but Welzel (2021) assumes cognitive mobilization comes first (p. 999).

To tie up the discussion of emancipative values with the concept of democratic deterrence, Welzel (2021) notes that the the lack of emancipative values will cause people lacking audacity to resist authoritarian indoctrination, such as ‘democracy redefinition’, which is currently quite trending, wherein democracy is redefined as sole obedience to the rules ^8^. Observing the rise of emancipative values, Welzel strongly states: “These observations reinforce the conclusion that strong emancipative values provide the mightiest antidote against authoritarian redefinition of democracy” (p. 1006).

## Optimism with caution on Indonesia

This section asks the following question: Can Indonesia be a part of the democratic deterrent of middle powers? If so, how can it play a role? This study argues that Indonesia has the potential to be just that. However, we must first assess its state of domestic democratization and its great-power rivalry position before we delve into its potential to advance democratic advantage narrative, especially in the area of the compatibility of Islam and democracy, disaster management, and climate change. During the most recent annual presidential address to Parliament, a day before Independence Day, President Joko Widodo with seeming pride quoting the crowning of middle-power status to Indonesia by an Australian think tank, that will result in Indonesia’s voice be heard more seriously on the international stage ^9^. Theoretically, middle powers do not influence the region powerfully as great powers that possess roles such as enforcers, hedgers and balancers. Middle powers are expected to play more the role of ‘legitimizers’ and ‘bridgers.’ ( Soeya, 2020, p. 10) The legitimizer role refers to the ability of middle power to convince the legitimacy of certain issues of its respective expertise to the top-tier and lower-tier powers. Bridger’s role, as its name suggests, refers to the ability of middle powers to play the role of an honest broker between two competing parties or groups.

At the G20 Summit in Bali, President Joko Widodo gained praise as an honest power broker between China and the United States when the relationship between those two great powers was strained due to trade wars, Nancy Pelosi’s visit to Taiwan, and Beijing’s ambivalent position on Russian’s invasion of Ukraine ^10^. During the speech, President Joko Widodo unveiled his effort to get everyone together in the same room and warned the world not to fall into another Cold War ^11^. In 2008, a notable breakthrough in Indonesian foreign policy in relation to democracy promotion occured in the form of the Bali Democracy Forum (BDF), a regional multilateral forum in which every state participant shares its own experience with democracy ( Rosyidin & Kusumawardhana, 2023, p. 10). BDF heralds a novel non-Western approach to promote democracy and reflects Indonesia’s role as a ‘bridge builder’ by “… bridging the perception gap towards democracy” (p. 12). The BDF has been an annual routine for Ministry of Foreign Affairs under the leadership of President Joko Widodo, even described as maintained with higher commitment than previous government did (p. 12).

Jakarta also played a significant role in formulating the ASEAN Charter, which was established to apply the rules-based order in the region, and made significant efforts to incorporate human rights and democratic principles into the charter. Unfortunately, several fellow members of ASEAN rejected the attempt, resulting in a less-than-optimal version of the final document ( Teo, 2021, p. 1151) ^12^. Umar (2023, p. 1467) explains how Indonesia “… embarked on initiatives for the introduction of universal human rights and democracy as key regional values for ASEAN”. Kleinfeld et al. (2021) describes middle power democracy as “… encompasses democratic countries (other than the United States) that have made supporting democracy a part of their foreign policy in a sustained manner, committing a nontrivial amount of diplomatic capital and/or aid resources to the issue on an ongoing basis”. Judging Indonesia’s ‘non-trivial’ commitment in BDF and ASEAN Charter, it is no stretch of imagination to see it as a promising middle-power democracy.

Indonesia’s projection of democracy within ASEAN explains volume about its foreign policy, considering the concept of ASEAN Centrality within its foreign policy ^13^. However, to assess Indonesia’s potential to be a middle-power democratic deterrence, we need to examine its state of democracy. Crouch (2010) offers a picture of Indonesian democracy with a series of political reforms following the fall of Soeharto in 1998. He analyzes reforms that occurred within institutions such as political parties, civil society organizations, and the military, along with discussions of political actors responsible for determining the direction of democratic change. From Crouch (2010)’s work we can see that Indonesia has achieved so much democratic progress: constitutional reform, direct presidential elections, decentralization, army out of the politics, among others; thus, no wonder Kishore Mahbubani calls its democratic progress a miracle ^14^, even though Crouch also discusses some points of fragility in the form of endemic corruption, the rise of identity politics, and the potential of communal conflicts.

Power & Warburton (2020) conclude that during the Presidency of Joko Widodo, Indonesia has suffered democratic regression due to populist mobilization, deepening sectarianism, deterioration of civil liberties, growing intolerance, executive’s expansion of an authoritarian toolkit for suppressing opposition and curtailing criticism, and dysfunctional electoral and representative institutions (pp. 1-2). Further, they divide the regression into two helpful categories: 1) democratic regression from above and 2) democratic regression from below. What is meant by democratic regression from above is the attack on democratic consolidation from actors and institutions, such as political party leaders, elected officials, state officials, and wealthy capitalists (p. 8). Regression from below refers to erosion in actors and institutions such as civil societies, NGOs, local leaders, and intellectuals, combined with declining protection of minorities, and growing polarization. Covid-19 pandemic made things worse by the growing practice of illiberal policies.

Going back to Welzel (2021)’s finding that the emancipative values determine the strength of democratization, according to the latest Wave 7 of the World Values Survey ^15^, Indonesia’s overall emancipative values score is worryingly lower than its regional neighbours of Thailand, Malaysia and Singapore, even though Indonesia has a higher score on the 5 high-level V-Dem Democracy indices ^16^ in 2023, compared to the three neighbours. Indonesia’s emancipative values score was even lower than that of China. Perhaps, the above findings are in concord with the findings of Asian Barometer survey that shows Indonesian public has an illiberal interpretation of democracy ( Warburton & Aspinall, 2019); and in concord with assessment of Fossati & Coma (2020, p. 186) that Indonesian public’s support of democracy is contigent with economic outcomes. If we are to believe the words of Diamond (2010, p. 37) that “… democratic stability depends heavily on robust public support for democracy” then this calls for caution on the future of Indonesian democracy. This caution is more justified if we consider the growing link between public-opinion-based domestic policy with foreign policy in Southeast Asia ( Murphy, 2017).

It is not easy to assess Indonesian “great-power-rivalry status,” as it claims to be non-aligned, since the non-alignment principle is one of the core pillars of its foreign policy. Iksan & Soong (2023, p. 317) decribe Indonesia’s hedging strategy towards great-power rivalry and conclude that Indonesia is tending towards the United States in politics and security affairs, while tending towards China in economics and investment affairs. Ardhani, Nandyatama, & Alfian, (2023) deems Indonesia’s promotion of the ASEAN Outlook on the Indo-Pacific (AOIP) as a response to US-China great power rivalry that asserts ASEAN neutrality and autonomy. Umar (2023, p. 1476) studies Indonesia’s engagement with the liberal international order (LIO) and observes how “… Asian middle powers attempt to navigate and manage great power politics as a part of their conceptions of international order,” and concludes with different vision of international order between the presidency of Yudhoyono and Joko Widodo, where the projection of democratic values is emphasized in the former, while the latter emphasizes economic development. Rosyidin & Kusumawardhana (2023) conclude that Indonesia’s current BDF endeavors is neither promoting nor projecting democracy. Khoo (2022) describes the ambivalence attitude of Southeast Asian states towards great power rivalry between U.S. and China, and that they are more interested in increasing their leverage. Murphy (2017) assesses Indonesia strategy towards great power rivalry as ‘hedging’ as opposed to Philippine’s ‘bandwagoning’ with China. Teo (2022) describes Indonesia’s bridging approach to great power rivalry as becoming a “good regional citizenship.” It is also interesting to study Indonesia’s attitude towards AUKUS, the three-way security pact between Australia, the United Kingdom, and the United States, that ranges from complaints ( Khoo, 2022, p. 309) to tacit acceptance ( Syailendra & Sebastian, 2021).

If Scandinavian countries are known for their expertise on climate change management and Singapore is known for its tradecraft in maritime and international law, Indonesia should hone its skill in certain specialty areas. As stated above, Indonesia has the potential to advance democratic advantage narrative especially in the areas of compatibility of Islam and democracy, disaster management, and climate change. Kleinfeld et al. (2021) describe the current era’s demand for democracy, that is, democracy must been seen as being able to ‘deliver’. Perhaps one cannot judge too harshly President Joko Widodo’s handling of BDF compared to President Susilo Bambang Yudhoyono’s, which prioritizes economic development over promotion of democracy, since democracy that does not deliver will doom to fail. Indonesia’s Nadhalatul Ulama (NU) is the world’s largest Islamic organization that boasting hundreds of millions of followers. In the group’s centenary celebration, attended by the President and top political figures, NU formally calls for the abandonment of the caliphate and accepts the nation state system. It has previously called on Muslims to reject viewing other religion as *kafir* or infidel, and one of its senior officials blatantly rejects *sharia* law practice as a positive law. Thus the recent headline of one of The Economist articles says, “Indonesia wants to export moderate Islam” ( The Economist, 2023). Abdurrahman Wahid, known as ‘Gus Dur’, the founder of NU, is a rare Islamic figures who believes in the universality of human rights based on Islamic teachings and advances it. He believed that the principle of universal humanity inheres in Islamic doctrine of *maqashid sharia* ( Supriyanto, 2018) *.* The belief in the universality of human rights by the founder of the largest Islamic organization in the world is by no means a small feat since the dominant view of human rights by most Muslim clerics in Indonesia is ‘cultural relativism’ ( Fuad, et al., 2007, p. 259).

Indonesia is seen as a nation with competence in disaster management. President Susilo Bambang Yudhoyono was named “UN Global Champion for Disaster Risk Reduction” and USAID official approves Indonesia superiority for disaster management ( Quayle, 2018, p. 141). It is one of the most disaster-prone countries in the globe, placed within the Pacific “Ring of Fire” and home to 76 active volcanoes. Since 2004 Indian Ocean Tsunami that took hundreds of thousand Indonesian lives, it overhauled its disaster management system and creating National Agency for Disaster Management in 2008. It also earned kudos for its good environmental performance, judged by its ratification of the Kyoto protocol, hosting of the UN Climate Conference in Bali, voluntary commitment to reduce greenhouse gas emissions, and various introductions of environmental laws (p. 143).

Kroenig (2020, p. 219) compares the share of global GDP of United States and China, respectively at 24% and 15% and concludes: “That is too close for comfort.” However, if one adds formal treaty allies and other world democracies to the U.S. side of the ledger, it is 75% share of world GDP. Again, President Joko Widodo’s focus on economic development in his BDF priorities should not be seen as less commitment to democracy but instead as emphasis on democracy that ‘delivers.’ Democracy must be seen as “the winning side” if it is to gain more traction in winning more countries to its side of the ledger.

One global trend that can be seen as positive for the democratic advantage narrative is the increasing sign of China’s model decline. Houze Song, an economist, concludes that China quietly abandoned its ambition to take over U.S.’ economy based on the observation of CCP’s 20 ^th^ National Congress ( Newsweek, 2022, p. 24). So much for China’s meritocracy hailed by Bell (2015), autocracy and blind loyalty made a comeback since President Xi Jinping removed the presidency time limit in 2018 to poised himself to rule indefinitely. Recent coverage of The Economist on China’s second-quarter economy shows a bleak picture of 3.2% growth whereas U.S. is predicted to grow 6%. Indicators such as house prices, consumer spending, business investment, and exports fall short and deflation is looming. Policy blunders such as excess infrastructure-building and stimulation of property markets by cheapening credit, mercantilist industrial policy, 2020-crackdown on technology firms, and lockdown mishandling, are cited as the reasons. The verdict: “Many of its challenges stem from broader failures of its economic policy-making—which are getting worse as President Xi centralises power.” ( The Economist, 2023b, p. 9).

## Indonesia’s Contribution to Democratic Deterrence in Great Power Rivalry

The return of great power rivalry has reignited the ideological contest between democracy and autocracy. As authoritarian powers increasingly challenge the liberal international order, they employ not only military and economic coercion but also sophisticated ideological narratives designed to undermine the legitimacy of democratic governance. In this context, the Republic of Indonesia—as the world’s third-largest democracy and largest Muslim-majority nation—occupies a pivotal strategic position. By demonstrating the compatibility of Islam with democratic norms through indigenous frameworks like *Maqashid Sharia*, Indonesia provides a powerful counter-narrative to authoritarian propaganda. This essay argues that Indonesia’s ideological example contributes to “democratic deterrence,” invalidating claims that liberal democracy is hostile to traditional values and thereby strengthening the “democratic advantage” in the contemporary geopolitical landscape.

To understand the strategic value of Indonesia’s example, one must first recognize the nature of the ideological threat it counters. Vladimir Putin has spearheaded a concerted effort to frame liberal democracy as inherently hostile to religious and traditional life, positioning authoritarianism as the necessary guardian of moral order. This narrative is not merely rhetoric but a core component of a geopolitical strategy to fracture the democratic world. Putin explicitly argues that the liberal democratic order threatens the cultural and spiritual foundations of society: “The liberal idea has become obsolete. It has come into conflict with the interests of the overwhelming majority of the population” He further contends that liberalism “presupposes that nothing needs to be done... that migrants can kill, plunder and rape with impunity because their rights as migrants have to be protected” ( Financial Times, 2019).

This propaganda seeks to delegitimize Western democratic models by framing them as existential threats to the “core population.” Putin asserts that liberalism “must not be allowed to overshadow the culture, traditions and traditional family values of millions of people making up the core population” ( Financial Times, 2019) Scholarly analysis confirms that this “vocally opposes the third liberal cultural script and denounces the post-1960s morality turn that has liberalised attitudes toward religion, family, gender, and sexual relations” ( Laruelle, 2022, p. 313). By doing so, “illiberalism... calls for a shift from politics to culture” (p. 309), attempting to displace the universal language of human rights with a particularist language of cultural rootedness.

Furthermore, this strategy involves the instrumentalization of religion to enforce political unity and reject democratic pluralism. In the Russian context, “Russian is Orthodox and Orthodox is Russian... The first proposition provides cover from external domination; the second proposition coaxes unity... Religion, it turns out, can be managed as well as democracy in the third way” ( Laruelle, 2009, p. 215). This effectively creates an ideological shield, where “Putin’s Valdai Club speech of 19 September 2013 was one of his most considered ‘ideological’ statements, presenting Russia as the keeper of a Western tradition that he argued the West itself had lost” ( Sakwa, 2017, p. 125). This narrative positions Russia “as a moral bastion against the decadence, sexual licence, porn and gay rights of the West” ( Sakwa, 2017, p. 127).

This ideological offensive extends to the Muslim world, where the Kremlin attempts to co-opt “traditional Islam” as a tool for state stability, framing it in opposition to foreign democratic influences. Moscow has helped to “revive the tradition of Tatar Islamic theology and stem the flow of students from Russia to the more radicalized Islamic schools in the Arab world and Pakistan” ( Trenin, 2018, p. 104). By cultivating reliance on authoritarian strongmen, such as Chechen leader Ramzan Kadyrov, who was “particularly helpful in the effort of presenting Russia’s Syria campaign as a war on terrorists who had abandoned true Islam” (p. 104), the Kremlin promotes a model of authoritarian stability over democratic accountability.

Against this backdrop of hybrid ideological interference, the concept of “democratic deterrence” becomes critical. Democratic deterrence posits that the very values authoritarian regimes attack—openness, pluralism, and accountability—can be leveraged as strategic assets. “Democratic deterrence shows how liberal democratic values need not be security vulnerabilities, but can be turned into strengths and tools to credibly deter hybrid aggressors, while making our Western democracies more robust and resilient” ( Wigell, 2021, p. 50). Rather than retreating into illiberalism, “Democratic deterrence focuses on strengthening our liberal democratic values and infrastructure... [It] is designed to render hybrid interference less efficient and less attractive as a strategy” (p. 64).

Indonesia contributes decisively to this form of deterrence by dismantling the core authoritarian argument that democracy is incompatible with religious values. Indonesia anchors its democratic practice in *Maqashid Sharia* (the higher objectives of Islamic law), offering a powerful intellectual proof that Islamic values are inherently aligned with universal human rights. “In Gus Dur’s reading, the five universal principles in the *maqashid syariah* discourse all lead to universal human interests” ( Supriyanto, 2018, p. 25). This framework refutes the notion that human rights are a Western imposition. Instead, “The principle of universal humanity as one of the universal teachings of Islam, in the view of Gus Dur actually exists in the treasures of Islamic thought, namely *maqashid sharia*” (p. 24).

By translating Islamic legal concepts into democratic imperatives, Indonesia demonstrates that “Government based on law, equality and tolerance towards different views are the main elements of humanity and, thus, display the universality of Islamic teachings” ( Supriyanto, 2018, p. 26). Specifically, the principles of *Maqashid Sharia* protect “The physical safety of the citizens... Safety of their respective religious beliefs... Family and family safety... Safety of property... and... Safety of property rights and professions” (p. 24-25). This synthesis creates a robust ideological immunity against authoritarian narratives that seek to pit faith against freedom. As former President Susilo Bambang Yudhoyono stated, “We in Indonesia have shown that Islam, democracy, and modernity can grow together” ( Rosyidin & Kusumawardhana, 2023, p. 9).

Indonesia’s successful synthesis of Islam and democracy contributes to a broader “democratic advantage” in the context of great power rivalry. Contrary to the belief that autocracies are more efficient, historical analysis suggests that “Democracies enjoy a built-in advantage in long-run great power competitions” ( Kroenig, 2020, p. 3). This is because according to Kroenig, states that excel in key economic, diplomatic, and military matters will perform better than states that do not and democracies appear to perform better in a range of key areas. By maintaining its democratic trajectory, Indonesia not only strengthens its own resilience but also validates the democratic model for other emerging powers.

Furthermore, Indonesia’s middle-power diplomacy amplifies this effect. In an era where “Western democracy still commands widespread attraction and political legitimacy” ( Wigell, 2021, p. 50), middle powers play a crucial role in renovating global democracy support. They often employ a strategy of focusing on “democracy-adjacent” issues to build coalitions. “Middle-power democracies generally prefer a soft and often indirect approach to supporting democracy. Rather than pursuing democracy as an ideological objective in and of itself, they like to link it with other values and goals perceived to be universal but less ideological … ” ( Kleinfeld, 2021, p. 13).

By engaging on issues such as anti-corruption and religious tolerance—framed through local contexts like *Maqashid Sharia*—Indonesia can reach audiences that might otherwise be skeptical of Western-led democracy promotion. “Middle-power democracies should direct their activity toward issues that have risen to the top of the global agenda but are adjacent to traditional democracy support” ( Kleinfeld, 2021, p. 21). This approach is particularly effective in swaying “swing states” in the Global South, which may be able to leverage greater concessions (p. 2) when backed by the solidarity of other democracies.

The ideological contest of the twenty-first century requires more than military strength; it demands the demonstration that democracy can deliver security, dignity, and continuity with tradition. Putin’s propaganda relies on the assertion that liberal democracy destroys traditional values. Indonesia’s democratic experiment, allied with the Islamic intellectual tradition of *Maqashid Sharia*, serves as a vital counter-proof. By showing that “Wielded with confidence, democracy itself is a potent strategic weapon” ( Wigell, 2021, p. 50), Indonesia contributes to a form of unique democratic deterrence that inoculates societies against authoritarian subversion. As great power rivalry intensifies, “Middle-power democracies have a crucial role to play in this generational challenge” ( Kleinfeld, 2021, p. 21), ensuring that the narrative of incompatibility between faith and freedom is exposed as false.

## Recommendations

Middle powers are known for being pragmatic. They are known to have utilized a hedging strategy in global-power rivalry, using the advantage of the rivalry to obtain certain goals. The Economist calls 25 non-aligned largest economies as “transactional 25” or T25, which includes India and Indonesia. They are deemed to be middle powers as “these middle powers are pragmatic and opportunistic.” ( The Economist, 2022b, pp. 50-52) However, the ideological nature of great power rivalry cannot be overlooked. While being transactional is not a sin, Indonesia has to use its ‘non-aligned democracy’ status to help secure world peace. While middle powers are known to be avoiding direct confrontation in countering authoritarianism ( Kleinfeld et al, 2021, p. 2), and in any case World War III must be avoided, it is necessary for democracies to deter authoritarianism for the sake of peace as Latin expression has it “ *Si Vis Pacem Para Belum*” meaning “if you want peace, prepare for war.”

Immanuel Kant introduces two types of peace in his book “Perpetual Peace.” The first type is the peace between democratic states that tends towards pacifism. The second type is peace between democracy and the non-democratic state which is “peace through deterrence.” ( Kant, 1795) As long as there is an authoritarian great power, democracy must always be on guard to deter. Indonesia, as a middle-power, non-aligned democracy, can play a unique role in this deterrence effort. Being non-aligned also has its advantage of being an honest power broker between the great powers, but it is naïve to overlook the aggressive tendency of autocrats as examples of such behaviour abounds in history, and it is simply unwise to disregard the democratic advantage argument.

This study has generated three recommendations. First, given Welzel (2021)’s findings on the centrality of emancipative values, we can argue that the most concerning threat to Indonesia’s democratization is its low emancipative values. Considering the increasingly intertwined nature of domestic public opinion and foreign policy in Southeast Asia, the public’s low emancipative values in Indonesia have become even more worrying ( Murphy, 2017). As noted above, emancipative values are preconditioned by cognitive mobilization and enabling living conditions. Therefore it is a ‘no-brainer’ to recommend more serious economic reforms or improve education system in Indonesia. For example, the World Bank recommends some education policy reforms to Indonesia’s government, such as improving teacher data accuracy, increasing coordination and communication among relevant ministries and local governments, implementing a performance-based recruitment system for civil servant teachers, standardizing non-civil service teacher recruitment, and improving teacher deployment to ensure more equitable education quality distribution ^17^. However, to add to the World Bank’s recommendation, it is important to focus on gender parity in educational reforms to combat learning inequalities in the country. The education of democratic values and human rights in the country must make religions as allies because Indonesia sees itself as a religious nation-state and acknowledges six official religions. Bell (1996, p. 652) argues that in East Asia context, failure to utilize religion in promoting human rights “… may lead politically moderate religious persons into developing feelings hostile to human rights positions”. Development of the theology of human rights in every recognized religion in Indonesia could be a potent way to accelerate human rights promotions, such as Waldron (2010)’s *Imago Dei* as a Christian theological basis for human rights or the aforementined Islamic doctrine of *maqashid sharia* as the basis for the universality of human rights in the eyes of Gus Dur, the founder of NU.

Second, Indonesia’s foreign policy needs to be more conscious of the democratic advantage argument, especially considering its status as the world’s third-largest democracy. Four traditional pillars of its foreign policy: non-alignment, attachment to UN’s principles, ASEAN Centrality and archipelagic outlook (Wawasan Nusantara) are not at all at odds with democratic advantage arguments, especially considering its active promotion of democratic values and human rights through ASEAN Charter and BDF initiative. As oxymoronic as it may sound, the status of a non-aligned democracy has its potential advantage as an honest power broker in times of conflict, as stated above ^18^. Even though Indonesia has a non-aligned foreign policy, Syailendra & Sebastian (2021) relate to how Indonesia’s “middle way” may be untenable and when push comes to shove in great power rivalry, it will eventually need to choose side. This brings us to the third recommendation.

Third, the West in general, and the United States in particular, need to be more supportive and appreciative of Indonesia’s democracy promotion efforts. Indonesia promotion of “Indo-Pacific Wide Friendship and Cooperation Treaty” based on ‘Bali Principles’ (principles that include respect for fundamental freedom and promotion of human rights) has not received U.S’ support and opposed by China that sees it as “… American plot to contain China” ( Ram, 2015, p. 25). As stated above, Indonesia is tending towards the United States in terms of the military, and to China in terms of economics ( Iksan & Soong, 2023). In other words, Washington remains Jakarta’s preferred defense partner. America needs to boost defense and security support for Indonesia. On August 24, 2023, U.S. Secretary of Defense, Llyod Austin and Indonesian Defense Minister released Joint Press Statement condeming China expansive maritime claims in the South China Sea and reaffirmed the importance of Indonesia’s military modernization and committed to deepen mutual interoperability “through defense capabilities like fighter aircraft upgrades, new multi-role fighter aircraft, and additional fixed and rotary wing transport aircraft” ^19^. Indonesia needs to defend its exclusive economic zone (EEZ) of the Natuna Sea from China’s claim of its as its sovereign territory within its nine-dashed line ( Burgess, 2023, p. 56). The recent purchase of 36 US F-15EX advance fighter aircraft can be seen as a good start in strengthening the defense cooperation between the two nations. More details on the need of ‘burden sharing’:

“US security cooperation with Indonesia is crucial for burden sharing and deterrence in the Natuna Sea, SCS, and eventually the wider Indo-Pacific region. As Beijing’s grand strategy aims to eventually dominate Eurasia and the Indo-Pacific, stronger partnerships and burden sharing are necessary for the United States to address the rising security challenge from China. Indonesia can contribute by developing greater capabilities for ISR and patrolling in the Natuna Sea and SCS. Meanwhile, the US military can focus on deterrence and denial tasks. To effectively deter China’s aggressive behavior in the region, the United States and Indonesia must commit to a division of labor in the event of PLA aggression in the Natuna Sea” (p. 73).

As European allies of NATO are deemed unable to defend their own continent since the Russian invasion of Ukraine, the United States is argued to be unprepared to fight on two major fronts simultaneously and is only equipped to defeat a single great-power adversary in one theatre ( Larsen, 2022, p. 10). China has developed sophisticated anti-access/area denial (A2/AD) capabilities to target critical nodes employed by the US Joint Force ( Kroenig, 2021). Kristiansen and Hoem (2022) make the case that it pays to be a security ally of NATO in the form of Security Sector Assistance (SSA), which can be a tool for achieving small states’ strategic objectives in an indirect manner, while also increasing its influence in the region. Giving Norway as an example, “contributing to an SSA-effort will include actually contributing to increased security locally, but also strengthening the alliance, ensuring increased deterrence – and more” (p. 412).

The United States’ response to Belt and Road Initiatives (BRI) with “Build Back Better World” and its successor the “Partnership for Global Infrastructure and Investment” so far are seen as insufficient, as America has to put more effort into leveraging its private business than China. As the Indo-Pacific Economic Framework (IPEF) includes cooperation on technological innovation, Indonesia should leverage this framework to accelerate progress in its technological sector. United States should help development of Indonesia’s technology to follow ‘Taiwan Model’ as opposed to ‘China’s Model,’ wherein the utilization of technology is used strategically to advance democracy as exemplified in Taiwan’s “people-public-private partnership” where citizens, companies, governments, and civic-techies can work together to create platform that provide economic and technological solution ( The Economist, 2021, p. 75).

## Conclusion

Democratic deterrence must have a better goal than just hybrid attacks prevention. Middle power democracy should have a higher goal than just to avoid World War III. Matthew Kroenig proposed a better plan for Washington to compete with China. Its goal is not to compete forever. The longer-term goal should be cooperative relationship with China. However, this would not happen as long as China is ruled by an aggressive autocratic regime. This leads us to the short-term goal:

“Washington needs to make China’s leadership understand that challenging the United States and its democratic allies is simply too difficult and too costly. Over time, Washington can convince Beijing that its own interests are better served by playing along with the rules-based system, rather than trying to challenge it” ( Foreign Policy, 2020).

According to Kroenig, this goal will not be easy, and it may take a generation or more. In other words, the goal is: “generational change of minds.” Democracies need to capitalize on their greatest strength: its open politics, free-market economy and innovative vibrancy, to deter autocrats until they change their minds and cooperate. Countries that find their place in this democratic deterrence effort will fulfil their destiny and be on the right side of history. Indonesia’s non-alignment policy does not forbid it from becoming part of deterrence for the democratic world, as Indonesia itself is a democracy.

## Data Availability

No data are associated with this article.

## Footnotes

^1^
For example see Acemoglu & Robinson (2013).

^2^
See Leeds (2003).

^3^
See Reiter & Stam (2002).

^4^

Jordaan (2003) for example distingushing between emerging and traditional middle powers with no reference to Indonesia yet acknowledging Malaysia despite Cooper (1997, p. 14) places Indonesia within first wave of new middle powers. Later works do place Indonesia as middle power such as: Umar (2023); Rosyidin & Kusumawardhana, (2023); Iksan & Soong, (2023); Ardhani, Nandyatama, & Alfian, (2023); Teo (2022) and Paris (2019). It is interesting to note Zala (2020, p. 72) refers to Indonesia as regional power and Quayle (2018, p. 134) went as far as stating: “The ‘great power’ within the regional international society of SEA is Indonesia. It is unfortunate that Kleinfeld, et al., (2021, p. 20) refers to Bali Democracy Forum without reference to Indonesia, while Umar (2023, p. 1460) refers to Indonesia as emerging “democratic middle power”. Moreover, according to Lowly Institute Asia Power Index, Indonesia is a middle power, see https://power.lowyinstitute.org/countries/indonesia/.

^5^
See https://www.pm.gc.ca/en/news/backgrounders/2022/06/10/canadas-new-investments-and-assistance-latin-america-and-caribbean.

^6^
The idea that governments are entitled to set their own rules for governing the internet even if those policies run afoul of international human rights principles ( Kleinfeld et al., 2021).

^7^

http://www.usindo.org/wp-content/uploads/2013/04/Open_Forum_Brief_Kliman_Fontaine_7.24.12.pdf.

^8^
See for example: https://www.businessinsider.com/china-whole-process-democracy-expert-explainer-mind-game-2022-10 where China’s White Paper describes its democracy as: “whole-process people’s democracy that integrates process-oriented democracy with results-oriented democracy” while one Oxford professor depicts it more as “Consultative Leninism”.

^9^
See https://nasional.kompas.com/read/2023/08/16/12571511/kutip-lembaga-australia-jokowi-sebut-indonesia-negara-middle-power-di-asia.

^10^

https://www.straitstimes.com/asia/se-asia/indonesia-s-jokowi-emerges-as-surprise-power-broker-at-g-20.

^11^

https://www.scmp.com/week-asia/politics/article/3199657/indonesias-jokowi-tells-g20-it-cannot-fail-another-cold-war-looms-world-divided .

^12^
Also see Umar (2023, p. 1470) where fundamental freedom is added besides democracy and human rights as the basis of the Charter, where the late former Foreign Minister, Ali Alatas described as having the determining role.

^13^

https://asean2023.id/en/news/asean-centrality-what-does-it-mean#:~:text=The%20ASEAN%20centrality%20concept%2C%20as,and%20engage%20with%20external%20powers.

^14^
See https://www.japantimes.co.jp/opinion/2008/09/22/commentary/world-commentary/indonesias-miraculous-free-democracy/.

^15^
Data can be accessed at https://www.worldvaluessurvey.org/WVSOnline.jsp.

^16^

https://www.v-dem.net/data/dataset-archive/.

^17^

https://www.worldbank.org/en/country/indonesia/brief/improving-teaching-and-learning-in-indonesia#:~:text=Policy%20recommendations%20to%20the%20government,service%20teacher%20recruitment%2C%20and%20improving.

^18^
See Gilley (2012) discusses non-aligned diplomacy of middle powers can “…influence the rise of China in ways that the United States cannot” in https://www.nytimes.com/2012/09/11/opinion/the-rise-of-the-middle-powers.html.

^19^
See https://www.defense.gov/News/Releases/Release/Article/3504476/united-states-dod-and-indonesia-mod-joint-press-statement/.
