# Supplementary material for: Democratic Deterrence of Middle Powers in Great Power Rivalry: The Case for Indonesia
Source: F1000Res. 2025 Dec 31;14:876. Originally published 2025 Sep 5. [Version 2] doi: 10.12688/f1000research.169856.2 (PMC12796793; doi:10.12688/f1000research.169856.2)
Supplement: Peer review history

# Reviewer report: Reviewer response for version 2

**DOI:** 10.5256/f1000research.194592.r446552

**Published:** 2026-02-09

**Authors:** Andreas Kruck

**Affiliations:** 1 Geschwister‑Scholl‑Institut für Politikwissenschaft (GSI), Ludwig‑Maximilians‑Universität München, München, Germany

Thank you very much for the opportunity to comment on the revised version of the manuscript, which contains a newly written section on "Indonesia’s Contribution to Democratic Deterrence in Great Power Rivalry".

 This new section argues that Indonesia can contribute to the "ideological contest between democracy and autocracy" by "demonstrating the compatibility of Islam with democratic norms through indigenous frameworks". Thereby, "Indonesia provides a powerful counter-narrative to authoritarian propaganda, since "Indonesia’s ideological example contributes to “democratic deterrence,” invalidating claims that liberal democracy is hostile to traditional values and thereby strengthening the “democratic advantage” in the contemporary geopolitical landscape."

 I appreciate the author's efforts and think that the new section makes useful points. However, as the remainder of the paper has not been revised and the majority of my critical comments on the previous version have therefore remained unaddressed, the improvements are unfortunately insufficient to change my overall assessment of this paper.

Is the topic of the opinion article discussed accurately in the context of the current literature?

Partly

Are arguments sufficiently supported by evidence from the published literature?

No

Are all factual statements correct and adequately supported by citations?

No

Are the conclusions drawn balanced and justified on the basis of the presented arguments?

Partly

Reviewer Expertise:

(theories of) power rivalries in IR, emerging powers

I confirm that I have read this submission and believe that I have an appropriate level of expertise to state that I do not consider it to be of an acceptable scientific standard, for reasons outlined above.

# Reviewer report: Reviewer response for version 2

**DOI:** 10.5256/f1000research.194592.r446551

**Published:** 2026-01-12

**Authors:** Haans J. Freddy

**Affiliations:** 1 Madras Christian College, Tambaram, Tamil Nadu, India

I have reviewed the revised version of the article, and it is fine. I recommend the article for indexing.

Is the topic of the opinion article discussed accurately in the context of the current literature?

Partly

Are arguments sufficiently supported by evidence from the published literature?

Yes

Are all factual statements correct and adequately supported by citations?

Yes

Are the conclusions drawn balanced and justified on the basis of the presented arguments?

Partly

Reviewer Expertise:

NA

I confirm that I have read this submission and believe that I have an appropriate level of expertise to confirm that it is of an acceptable scientific standard.

# Reviewer report: Reviewer response for version 1

**DOI:** 10.5256/f1000research.187239.r415711

**Published:** 2025-11-22

**Authors:** Haans J. Freddy

**Affiliations:** 1 Madras Christian College, Tambaram, Tamil Nadu, India

the authors make an importnat observation in the light of the present war between Russia and Ukraine and the doctrine of great power competition between democracy and autocracy. this is important as the present world as the decline of democracy is becoming more relevant and more and more countries are behaving in that manner.

 in para 3, the authors again make an interesting observation that the present day geopolitics is being again determined by ideological concerns and not just in terms of economic, military diplomatic or technological factors. 

 The authors although are making their points clear must check for grammatical errors for instance ... democracy survival... does not really fit in well with the sentence it could rather be ...survival of democracy...

 the authors further make their arguments using the work of Kroenig to argue against autocracy and present democracy as a better model which is substantiated well

 overall the arguments presented are well justified. the style of presentation has a flow in it. however, the authors devote very less space for presenting the Indonesia as a nation which can paly a role in democratic deterrence. questions such as how Indonesia is a middle power and how it can play in strengthening democracy in international affairs will be more useful. Second Indonesia as a middle power what can be its contribution to great powers who are democratic will be significant which this article does not really make much reference to. Third, How can Indonesia as a middle power contribute towards democratic deterrence in great power rivalry must be given more attention as that is the title of the article. This is to say the Article spends more time in making a case for great power rivalries based on ideological concerns rather than presenting a strong case for Indonesia to be a middle power that has the ability to contribute to democratic deterrence and how it can.

Is the topic of the opinion article discussed accurately in the context of the current literature?

Partly

Are arguments sufficiently supported by evidence from the published literature?

Yes

Are all factual statements correct and adequately supported by citations?

Yes

Are the conclusions drawn balanced and justified on the basis of the presented arguments?

Partly

Reviewer Expertise:

International relations, War

I confirm that I have read this submission and believe that I have an appropriate level of expertise to confirm that it is of an acceptable scientific standard, however I have significant reservations, as outlined above.

# Response

**Published:** 2025-12-30

**Authors:** Aristo Purboadji

**Affiliations:** Theology, STT Tiberias Jakarta, Jakarta Selatan, DKI Jakarta, Indonesia

**Reviewer 1: Haans J. Freddy**

 We thank Reviewer Freddy for his encouraging and constructive feedback, as well as for his helpful suggestions on strengthening the manuscript’s analytical clarity and Indonesia-focused contribution.

**Comment :**

*The article spends more time on great power rivalry rather than presenting a strong case for Indonesia’s role. Questions such as how Indonesia qualifies as a middle power and how it can strengthen democracy in international affairs would be more useful.*

**Response:**

 We appreciate this comment and agree that Indonesia’s role needed to be articulated more explicitly. In response, we have **added a new dedicated section** titled **“Indonesia’s Contribution to Democratic Deterrence in Great Power Rivalry,”**placed after the section *“Optimism with Caution on Indonesia”* and before *“Recommendation.”*

 This new section explicitly answers *how* Indonesia, as a Muslim-majority democracy and middle power, contributes to great power rivalry through an **ideational mechanism**. Specifically, we argue that Indonesia’s experience demonstrates the compatibility of Islam and democracy and thereby counters authoritarian civilizational narratives—particularly those that relativise democracy and human rights by portraying them as incompatible with tradition and religion. We identify Indonesia’s use of *maqāṣhid sharīʿa* as the key mechanism through which democratic norms and universal human rights are legitimised from within Islamic jurisprudence.

 By framing this as an ideational contribution to **democratic deterrence**, the revised manuscript now clarifies Indonesia’s agency in international affairs rather than presenting it merely as a descriptive case.

**Comment :**

*The article would benefit from clarifying what Indonesia actually contributes to democratic deterrence beyond normative aspiration.*

**Response:**

 We have addressed this by explicitly identifying Indonesia’s contribution as **ideational rather than material or coercive**. The revised section demonstrates how Indonesia constrains authoritarian influence by reducing the plausibility of civilizational arguments that depict democracy as culturally Western or religiously incompatible. This directly strengthens democratic deterrence by narrowing the discursive space available to authoritarian challengers in global ideological competition.

# Reviewer report: Reviewer response for version 1

**DOI:** 10.5256/f1000research.187239.r429485

**Published:** 2025-11-20

**Authors:** Assad Mehmood Khan

**Affiliations:** 1 Minhaj University Lahore, Lahore, Punjab, Pakistan

The paper includes extensive citations and references to credible works (academic texts, policy documents, media). However, several claims require clearer empirical support:

- Assertions about *Indonesia’s low emancipative values relative to regional peers* should cite specific **WVS 2023 data tables or figures**.
- The mention of the *purchase of 36 F-15EX fighters* and defense cooperation updates (2023) rely on journalistic sources; cross-verification with official government releases would improve reliability.
- A few geopolitical generalizations (e.g., “many Chinese believe the American decline has finally come”) could benefit from empirical or survey data.

**Must address:**

- Add explicit data references (e.g., WVS dataset ID, Lowy Institute indices) and qualify journalistic or interpretive claims.
- Reinforce the Indonesia-specific analysis with data and empirical research on Indonesian civic education and Islamic democratisation.

Is the topic of the opinion article discussed accurately in the context of the current literature?

Yes

Are arguments sufficiently supported by evidence from the published literature?

Partly

Are all factual statements correct and adequately supported by citations?

Partly

Are the conclusions drawn balanced and justified on the basis of the presented arguments?

Yes

Reviewer Expertise:

IR, Political Dynamics

I confirm that I have read this submission and believe that I have an appropriate level of expertise to confirm that it is of an acceptable scientific standard.

# Response

**Published:** 2025-12-30

**Authors:** Aristo Purboadji

**Affiliations:** Theology, STT Tiberias Jakarta, Jakarta Selatan, DKI Jakarta, Indonesia

**Reviewer 2: Assad Mehmood Khan**

 We thank Reviewer Khan for his thoughtful engagement with the manuscript and for highlighting the need for greater analytical precision and balance.

**Comment:**

*Reinforce the Indonesia-specific analysis with data and empirical research on Indonesian civic education and Islamic democratisation.*

**Response:**

 We have added a new dedicated section, **“Indonesia’s Contribution to Democratic Deterrence in Great Power Rivalry.”** This section strengthens the manuscript by explicitly linking Indonesia’s Islamic civil society traditions—particularly the role of Islamic democratisation grounded in *maqāṣid al-sharīʿa*—to democratic norms and available empirical research on political socialisation and religious pluralism.

# Reviewer report: Reviewer response for version 1

**DOI:** 10.5256/f1000research.187239.r415715

**Published:** 2025-11-12

**Authors:** Andreas Kruck

**Affiliations:** 1 Geschwister‑Scholl‑Institut für Politikwissenschaft (GSI), Ludwig‑Maximilians‑Universität München, München, Germany

This article studies how middle powers can be parts of "democratic deterrence" in an era of great power rivalry and how Indonesia can "play a role in such deterrence". Based on recent publications by Kroenig (2020), Wigell (2021). Kleinfeld et al. (2021) and others, the author highlights the importance of "democratic deterrence" in contemporary power rivalries and points to several strategies and measures through which middle powers can contribute to democratic deterrence. While also acknowledging difficulties and limitations, the article overall underlines the potential of Indonesia to strengthen democratic deterrence.

 This article addresses a timely and important topic. It draws on policy-relevant conceptual and theoretical debates in International Relations (IR). It makes numerous important observations and plausible arguments.

 However, the article also has significant limits and weaknesses.

 1) Its conceptual, theoretical and empirical-analytical contributions are limited because the questions are too broad and the conceptual and theoretical statements in the article are too vague. I'd strongly suggest to focus conceptually and empirically on a limited set of strategies (and/or measures) through which middle powers can potentially enhance the resilience of democracies in great power rivalries. This limited set of selected strategies should then be conceptualized and empirically analyzed in much greater depth. As it stands, the article touches upon (too) many different aspects but fails to explicate its key points. Accordingly, the answers to the research questions also remain vague. Let me elaborate this overarching point in more specific detail below.

 2) The concept of democratic deterrence requires further elaboration. First, deterrence is a longstanding key concept in IR. Against this background, the reception of relevant literature on deterrence is very selective. Second, and more importantly, the more specific concept of democratic deterrence also needs clarification and further elaboration. E.g., it remained unclear to me when, why, and how democratic deterrence works and is successful. This would be key to later assess Indonesia's opportunities for engaging in it. Third, the article is, at least in parts, unclear with regard to what is supposed to be deterred? "Only" hybrid interference attacks, or also territorial military aggression? And who is the target of deterrence, "only" external autocratic (state) powers or also their intermediaries and/or domestic forces within democracies?

 3) The article seems to neglect or downplay developments in the "liberal West", including the US, which challenge key premises of the article. It rests on the assumption of a dichotomy between liberal powers led by the US and autocratic rivals led by China and Russia. However, it hardly problematizes internal illiberal trends in the "liberal West", including the US under Trump, which may question their liberalism and, by implication, the view of an ideological rivalry between liberal democracies and autocracies as the key threat to global democracy and peace.

 4) The article rests on strong normative commitments of the author. This is fine as such. However, I think the author should a) make even more transparent their own normative positionality in this debate and b) also consider potential undesired implications of discursively reinforcing the notion of current power rivalries amounting to an existential battle between liberal democracies and autocracies. Viewing manifold and diverse conflicts between, among others, the US and China in various issue areas merely as one big ideological struggle between democracy and autocracy risks to contribute to exacerbating the rivalry.

 5) In my analysis, there are commonalities but also significant differences in the postures, power and behavior of China and Russia - and other emerging authoritarian powers in the BRICS+. In my view, the article would benefit from a) differentiating more between China and Russia and b) bringing in other (regional) authoritarian rising powers, such as Saudi-Arabia, Turkey, etc. 

 6) I am not a country expert of Indonesia so I'll be cautious in my assessment of the final parts of the article. However, I do think the article would strongly benefit from a more extensive and nuanced assessment of the state of democracy in Indonesia. For example, V-Dem data show a decline in all major sub-indices for Indonesia democracy for the last 10 to 15 years. What does that mean for Indonesia's potential for "democratic deterrence"? 

 7) More fundamentally, I fail to see how Indonesia's posture as a non-aligned hedging country could be reconciled with taking a strong and proactive stance in the promotion of a liberal democratic advantage narrative. At the very least, this balancing act requires far more discussion and explanation. 

 8) A small empirical point: The rather negative assessment of China's capacities and prospects in technological rivalries (including AI, telecommunications governance etc.) seems insufficiently backed up by empirical evidence, while ignoring past evidence of and future possibilities for Chinese advancements.

Is the topic of the opinion article discussed accurately in the context of the current literature?

Partly

Are arguments sufficiently supported by evidence from the published literature?

No

Are all factual statements correct and adequately supported by citations?

No

Are the conclusions drawn balanced and justified on the basis of the presented arguments?

Partly

Reviewer Expertise:

(theories of) power rivalries in IR, emerging powers

I confirm that I have read this submission and believe that I have an appropriate level of expertise to state that I do not consider it to be of an acceptable scientific standard, for reasons outlined above.

# Response

**Published:** 2025-12-30

**Authors:** Aristo Purboadji

**Affiliations:** Theology, STT Tiberias Jakarta, Jakarta Selatan, DKI Jakarta, Indonesia

**Reviewer 3: Andreas Kruck**

 We thank Reviewer Kruck for his rigorous and critical engagement, which has helped us sharpen the manuscript’s conceptual clarity and scope.

**Comment :**

*The conceptual contribution of the article remains unclear, and the strategies discussed are too broad and insufficiently specified.*

**Response:**

 We have addressed this concern by **substantially narrowing and specifying the manuscript’s contribution**. In Version 2, we no longer present multiple loosely connected strategies. Instead, we focus explicitly on **one ideational mechanism**through which a middle power can contribute to democratic deterrence.

 The newly added section on Indonesia clarifies the conceptual contribution by showing how democratic deterrence operates at the ideational level, specifically through contestation over the universality of democracy and human rights in great power rivalry. This directly responds to the concern about conceptual vagueness.

**Comment :**

*The manuscript should clarify its normative positioning and avoid conflating analysis with advocacy.*

**Response:**

 We have taken care in Version 2 to distinguish **analytical explanation from normative endorsement**. While the manuscript recognises the normative value of democracy, the revised argument is framed analytically: Indonesia’s Islam–democracy compatibility is examined as a **strategic constraint on authoritarian narratives**, not as a moral appeal.

 By grounding the argument in documented ideological contestation and by using verbatim primary sources, the revised manuscript avoids advocacy and instead analyses how ideational power functions in great power rivalry.

**Concluding remark**

 We believe that the revisions in Version 2 have significantly strengthened the manuscript by clarifying Indonesia’s role, narrowing the analytical focus, and explicitly situating democratic deterrence within contemporary great power rivalry. We are grateful to all reviewers for their valuable feedback, which has substantially improved the quality and clarity of the article.
